# Perceived supervisor support and its impact to job performance: Dataset from the hospitality industry in Vietnam

**DOI:** 10.1016/j.dib.2025.111649

**Published:** 2025-05-13

**Authors:** Bao Han Le, Cong Hiep Duong, Gam Thi Nguyen

**Affiliations:** a Business Administration Faculty, Industrial University of Ho Chi Minh City, Viet Nam; b College of Management, Chang Jung Christian University, Taiwan; c Faculty of Accounting, Thai Nguyen University of Economics and Business Administration, Thai Nguyen, Viet Nam; d Faculty of Business and Economics, Phenikaa University, Yen Nghia, Ha Dong, Ha Noi, Viet Nam

**Keywords:** Hospitality industry, Perceived supervisor support, Job satisfaction, Organizational commitment, Job performance, Vietnam

## Abstract

The dataset explores the intricate relationship between Perceived Supervisor Support (PSS) and Employee Job Performance (JP) within Vietnamese hospitality industry. It highlights the critical influence of Job Satisfaction (JS) and Organizational Commitment (OC) against the backdrop of the country's socialist economic structure. A structured questionnaire was circulated among travel agencies in Northern Vietnam, collecting 322 valid responses via snowball sampling. Data were rigorously analyzed using Partial least squares structural equation modeling (PLS-SEM) to clarify how PSS, JS, OC, and JP interact. The data highlights the pivotal impact of PSS, JS, and OC on enhancing JP and provides strategic insights for policy makers to improve employee performance management in the Vietnam’s hospitality industry.

Specifications Table

**Table:** 

Subject	Business, Management, and Decision Science.
Specific subject area	Human Resource Management, Hospitality industry, Organizational Behaviour.
Type of data	Table, Figure. Raw.
Data collection	The data was gathered directly from September 2023 to March 2024 using the snowball sampling method. Participants completed a detailed questionnaire consisting of seven demographic questions and seventeen additional questions targeting Perceived Supervisor Support (PSS), Job Performance (JP), Job Satisfaction (JS), and Organizational Commitment (OC). Only individuals currently working in the hospitality sector and aged 18 or above were considered eligible for this study. Participants were briefed on the research's objectives and importance prior to answering the survey questions. A total of 480 Vietnamese hospitality workers who met the eligibility criteria were invited to participate through a questionnaire. Ultimately, 360 responses were collected, representing a response rate of 75%. After data cleaning, 322 valid responses were analyzed.
Data source location	Region: Northern of Vietnam Country: Vietnam
Data accessibility	Repository name: Mendeley Data Data identification number: 10.17632/c5dwcxwnyj.1 Direct URL to data: https://data.mendeley.com/datasets/c5dwcxwnyj/1 …
Related research article	*.*

## 1 Value of the Data

• The dataset underscores the significant impact of perceived supervisor support on job performance of employees in the hospitality industry in Vietnam, mediated by increases in organizational commitment and job satisfaction.
• A survey investigated the impact of Perceived Supervisor Support, Job Satisfaction, and Organizational Commitment on employees' Job Performance in the hospitality industry.
• Leaders and policy-makers in the hospitality industry can leverage the data analysis results from this study to enhance employee commitment and performance, ultimately driving customer satisfaction and business performance.
• As one of the first surveys on the impact of PSS on JP in the hospitality industry in Vietnam, this dataset is expected to inspire collaborations with researchers from developed countries or different industry. Such partnerships will facilitate the replication of this study and allow for a comparative analysis of the results.
• The findings from this data set reveal that perceived supervisor support significantly and positively influences job performance. This aligns with the results of a study conducted by Frear, Donsbach, Theilgard and Shanock [1] and among employees at USA government agency, and also consistent with a study in the telecommunications industry in Pakistan [2]. However, these findings inconsistent with the results of a research conducted in ten small to medium-sized industrial companies in Spain, research indicated that perceived supervisor support does not significantly impact job performance [3].

## 2 Background

The dataset provides a detailed examination of the relationships among PSS, JS, OC, and JP, specifically within Vietnamese hospitality industry. Prior to the Covid-19 pandemic, international tourism constituted approximately 8% of Vietnam's gross domestic product (GDP), with domestic tourism contributing roughly 4% [1]. Post-pandemic, the hospitality industry in Vietnam is undergoing a phase of recuperation. Reports for 2023 indicate that the nation will accommodate over 12.5 million international visitors and manage the influx of upwards of 108 million tourists, both domestic and international. Vietnamese tourism occupies the sixth position worldwide in terms of growth in search volumes originating from international markets [2]. The hospitality industry in Vietnam harbors substantial capacity for expansion, necessitating a framework for stable and sustainable growth. To this end, managerial staff are pivotal in directing employee efforts towards enhancing JP [3]

## 3 Data Description

From September 2023 to March 2024, the survey was executed in two stages. First, a pilot survey targeted 50 employees in the hospitality industry in Vietnam. This diverse group included participants from different roles and experience levels to ensure a comprehensive assessment of the clarity and relevance of the questionnaire to a wide range of employees. During the pilot phase, participants were asked to complete the questionnaire and provide feedback on the clarity of the questions and the appropriateness of the response scale. This feedback was critical in identifying any items that were ambiguous or misleading. We simplified the language of some questions that participants found difficult to understand, making them easier for employees at all levels to understand. We also made adjustments to ensure all questions were culturally appropriate and relevant to the specific context of the hospitality industry in Vietnam. Additionally, we conducted an initial analysis of the pilot data to assess the internal consistency of the questionnaire, which resulted in the lowest Cronbach's alpha and Composite reliability coefficients of 0.725 and 0.816, respectively, indicating a high level of reliability.

**Fig. 1:**
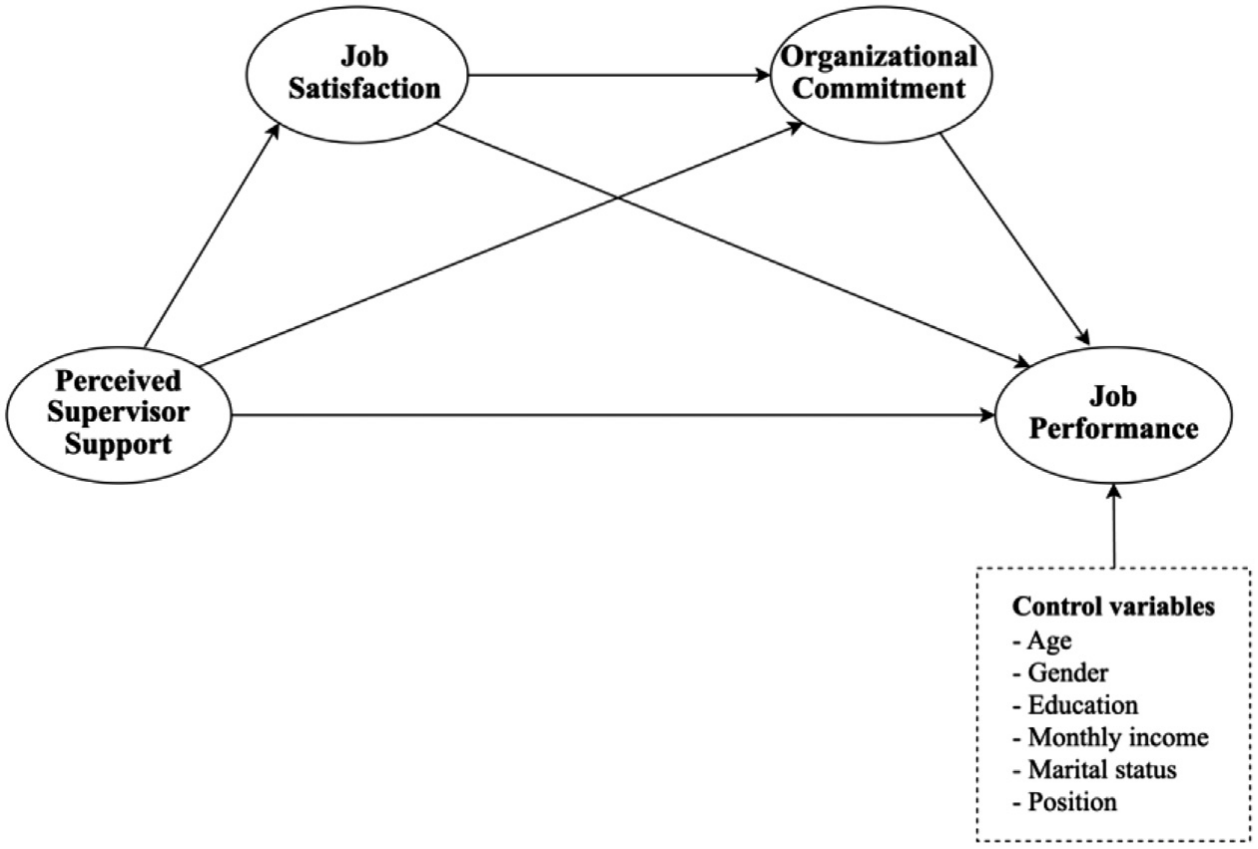
Conceptual model.

Second, a broader survey was conducted, accumulating 322 analyzable responses. The data are stored in two principal documents that delineate the data characteristics and its applications. Firstly, “Data-PSS-JP.csv,” includes numerical data from surveys of employees in Vietnam's hospitality industry, structured for statistical analysis and theoretical modeling and comprises 322 responses focusing on PSS, JS, OC, and JP. Secondly, “Questionnaire.docx” contains all the questions utilized in the data collection process. A total of 480 Vietnamese hotel industry employees who met the criteria were invited to participate in the survey via a questionnaire. The study population comprised full-time employees aged 18 years and above. Potential respondents were approached through both personal and professional channels. The author reached out to prospective participants via telephone to invite them to take part in the study. After receiving their consent, the survey questionnaire was administered in person. Participants were then asked to distribute the questionnaire to their colleagues across different departments within their organizations. Additionally, the author designated a representative from each company to assist with data collection and prevent duplicate responses from the same individual. Ultimately, 360 responses were collected, representing a 75% response rate. After cleaning the data by excluding incomplete responses to the questionnaire, 322 valid responses were analyzed. This survey addresses four main aspects: PSS, JS, OC, and JP, as depicted in Fig. 1.

Table 1 represents the demographic characteristics of respondents, the demographic data reveals that the age group 18-25 was predominantly represented, constituting 43.17% of participants, followed by the 26-36 age group at 34.16%, and a smaller proportion above 47 years at 4.35%. The survey data shows that females were the majority, accounting for 72.67% of participants, this indicates that the majority of the labor force in Vietnam's tourism industry is female, the finding aligns with the report from [4]. In terms of marital status, 25.78% of respondents were single, while 74.22% were married. The majority of respondents were employed as workers (57.45%), with leaders comprising 23.91%, head or deputy directors 14.91%, and a minority involved in festival boards or associations at 3.73%. ComprehensivSe details of the demographic distribution are presented in Table 1.

**Table 1:** Sample characteristis (N=322).

Code	Characteristics	Categories	N	(%)
Q1	Age	18-25	139	43.17
		26-36	110	34.16
		37-47	59	18.32
		> 47	14	4.35
Q2	Gender	Male	88	27.33
		Female	234	72.67
Q3	Education	High school and below	93	28.88
		University/College	198	61.49
		Master and above	31	9.63
Q4	Monthly income	Less than $300	43	13.35
		$301-$500	181	56.21
		$501-$700	85	26.4
		> $700	13	4.04
Q5	Marital status	Single	83	25.78
		Married	239	74.22
Q6	Position	Board of Directors	12	3.73
		Chief/Deputy Chief	48	14.91
		Leader	77	23.91
		Employee	185	57.45

*Note*: 1 USD, approximately 25,000 VND during the survey period.

Each factor in this research was meticulously adapted from prior research to align with the distinct attributes of this research. PSS was evaluated utilized a five-item scale modified from the work of Eisenberger, Stinglhamber, Vandenberghe, Sucharski and Rhoades [5]. Organizational Commitment (OC) was gauged through a fourth-item scale adapted from García-Rodríguez, Dorta-Afonso and González-De-la-Rosa [6]. Satisfaction (JS) was measured by a five-item scale based on the studies of Moqbel, Nevo and Kock [7]. Job Performance (JP) was assessed utilized a three-item scale developed from the study of Moqbel, Nevo and Kock [7]. A five-point Likert scale was used for all constructs in this research. These scale provide a comprehensive and nuanced framework for exploring the complex interactions among various factors influencing JP. Table 2 lists the specific items included in the questionnaire.

**Table 2:** Measurement scale.

Construct	Item	Description	Sources
Perceived supervisor support (PSS)	PSS1	My supervisor strongly considers my goals and values	Eisenberger, Stinglhamber, Vandenberghe, Sucharski and Rhoades [5].
	PSS2	My supervisor cares about my well-being	
	PSS3	My supervisor is willing to help me when I need a special favour	
	PSS4	My supervisor shows very little concern for me.	
	PSS5	My supervisor takes pride in my accomplishments at work	
Employee’s job performance (JP)	JP1	My performance in my current job is excellent	Moqbel, Nevo and Kock [7]
	JP2	I am very satisfied with my performance in my current job	
	JP3	I am very happy with my performance in current job	
Job satisfaction (JS)	JS1	I am very satisfied with my current job.	Moqbel, Nevo and Kock [7]
	JS2	My present job gives me internal satisfaction.	
	JS3	My job gives me a sense of fulfillment.	
	JS4	I am very pleased with my current job.	
	JS5	I will recommend this job to a friend if it is advertised/announced.	
Organizational Commitment (OC)	OC1	This organization deserves my loyalty	García-Rodríguez, Dorta-Afonso and González-De-la-Rosa [6]
	OC2	I feel a strong sense of belonging to this organization	
	OC3	I would be very happy to spend the rest of my career with this organization	
	OC4	I would recommend this hotel as a place to work	

Survey responses were evaluated using a 5-point Likert scale, ranging from “strongly disagree (1)” to “strongly agree (5)”, with mean scores between 4.21 and 4.33. We conducted detailed statistical analyses, which included calculating standard deviation and variance to examine response variability and distribution. Additionally, measures of skewness and kurtosis were calculated to understand the shape of the distribution. This comprehensive statistical analysis is crucial for interpreting response patterns and verifying the reliability of the measured constructs. Essential statistical metrics, including mean, standard deviation, skewness, and kurtosis, are presented in Table 3 and were analyzed using SPSS version 25

**Table 3:** Descriptive statistics of the constructs’ items.

Items	Mean	Std. Deviation	Skewness		Kurtosis	
			Statistic	Std. Error	Statistic	Std. Error
OC1	4.24	0.627	-0.299	0.136	-0.249	0.271
OC2	4.31	0.605	-0.359	0.136	-0.167	0.271
OC3	4.32	0.602	-0.368	0.136	-0.162	0.271
OC4	4.32	0.611	-0.468	0.136	0.254	0.271
PSS1	4.28	0.572	-0.083	0.136	-0.517	0.271
PSS2	4.32	0.612	-0.558	0.136	0.695	0.271
PSS3	4.31	0.592	-0.214	0.136	-0.609	0.271
PSS4	4.25	0.59	-0.121	0.136	-0.474	0.271
PSS5	4.24	0.603	-0.244	0.136	-0.087	0.271
JS1	4.3	0.588	-0.27	0.136	-0.074	0.271
JS2	4.33	0.593	-0.338	0.136	-0.129	0.271
JS3	4.31	0.61	-0.293	0.136	-0.638	0.271
JS4	4.21	0.561	0.018	0.136	-0.233	0.271
JS5	4.28	0.581	-0.123	0.136	-0.525	0.271
JP1	4.25	0.587	-0.118	0.136	-0.48	0.271
JP2	4.3	0.579	-0.148	0.136	-0.586	0.271
JP3	4.28	0.618	-0.337	0.136	-0.213	0.271

**Fig. 2:**
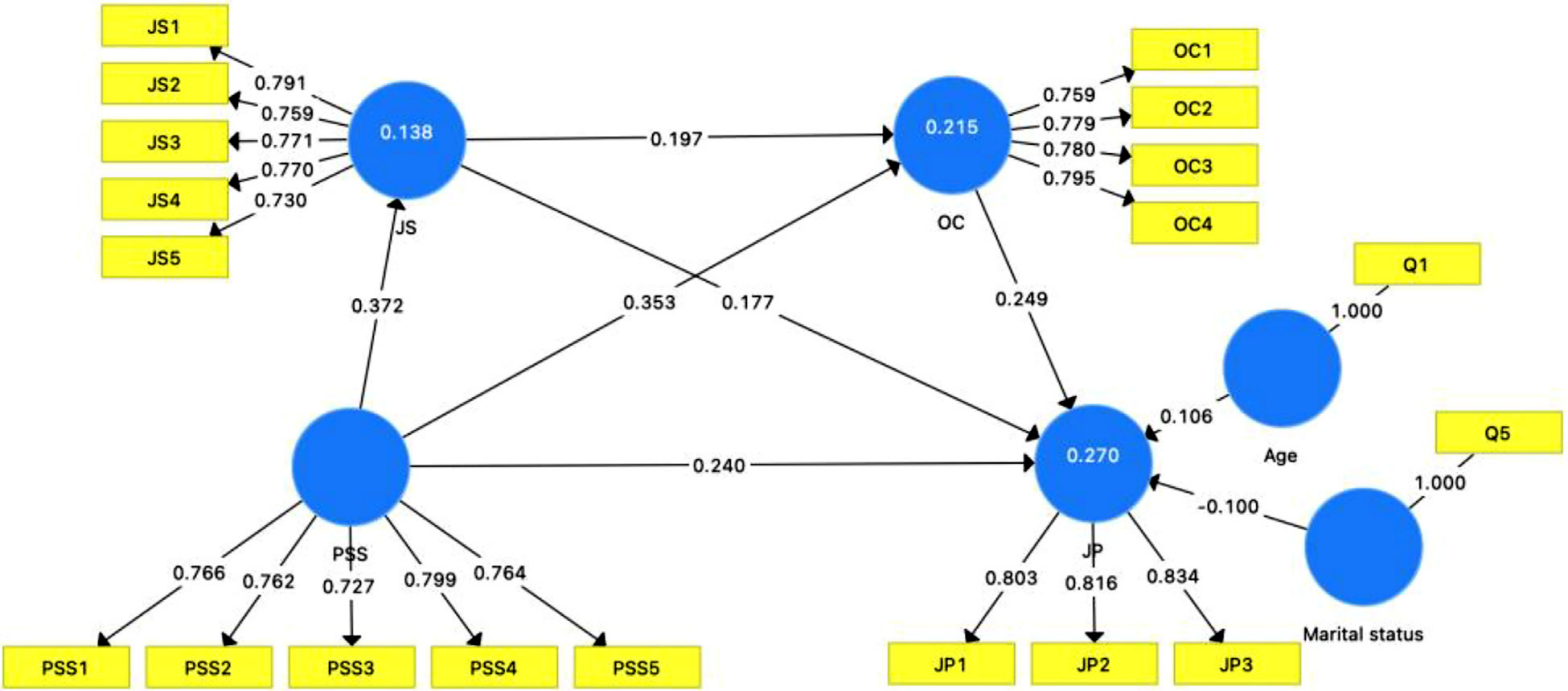
The PLS-SEM result.

The following tables contain key results for evaluation purposes. Table 4, Table 5, Table 6, Table 7 include the results of a series of statistical analyses that verify convergent validity , discriminant validity and internal consistency reliability of the measurement models and the explanatory and predictive power of the structural model [[8], [9], [10], [11], [12], [13], [14], [15], [16]]. Furthermore, Fig. 2 depicts the configuration of the measurement model used in the dataset.

**Table 4:** Reliability and convergent validity.

Items	Outer loading	VIF	CA	CR	AVE
PSS1	0.766	1.676	0.821	0.875	0.583
PSS2	0.762	1.655			
PSS3	0.727	1.479			
PSS4	0.799	1.765			
PSS5	0.764	1.555			
JP1	0.803	1.439	0.752	0.858	0.669
JP2	0.816	1.519			
JP3	0.834	1.599			
JS1	0.791	1.709	0.822	0.875	0.584
JS2	0.759	1.571			
JS3	0.771	1.656			
JS4	0.770	1.658			
JS5	0.730	1.504			
OC1	0.759	1.480	0.783	0.860	0.606
OC2	0.779	1.534			
OC3	0.780	1.580			
OC4	0.795	1.561

**Table 5:** Discriminant validity with Fornell-Larcker Criterion and HTMT Ratio.

Fornell-Larcker criterion				
	JP	JS	OC	PSS
JP	0.818			
JS	0.348	0.764		
OC	0.398	0.329	0.778	
PSS	0.408	0.372	0.426	0.764
Heterotrait-Monotrait Ratio (HTMT)				
	JP	JS	OC	PSS
JP				
JS	0.442			
OC	0.517	0.408		
PSS	0.518	0.453	0.526

**Table 6:** Path analysis.

Paths	f-square	Direct effects	Indirect effects	Total effects
PSS -> JP	0.060	0.240***		0.412***
PSS -> JS	0.161	0.372***		0.372***
PSS -> OC	0.136	0.353***		0.426***
JS -> JP	0.035	0.177**		0.226***
JS -> OC	0.043	0.197***		0.197***
OC -> JP	0.066	0.249***		0.249***
Age->JP		0.106*		0.106*
Marital status->JP		-0.100*		-0.100*
PSS -> JS -> JP			0.066**	
PSS -> OC -> JP			0.088**	
PSS -> JS -> OC -> JP			0.018**	

∗ p < 0.05; ∗∗ p < 0.01; ∗∗∗ p < 0.001.

**Table 7:** Structural model explanatory and predictive power.

Constructs	R^2^	Q^2^
PSS		
JP	0.270	0.158
JS	0.138	0.078
OC	0.215	0.123

## 4 Experimental Design, Materials and Methods

This study employed a quantitative methodology, building on previous research. Data was gathered using a structured survey questionnaire. Initially, a pilot test with 50 participants was conducted to assess the survey's structure and content. Based on the feedback from this initial phase, the survey questions in the Vietnamese version and the format of the questionnaire have been refined to enhance their effectiveness for the main data collection. The pilot participants reported clear understanding of the questionnaire items. The finalized questionnaire is divided into five sections: Demographics, PSS, JS, OC, and JP.

The questionnaire comprises two distinct sections: the first section solicits demographic data from the respondents, whereas the second section probes into principal variables under investigation. This includes the PSS and JS using five specific questions. OC is gauged through four items, and JP is assessed with three items. Responses were elicited on a five-point Likert scale, extending from “strongly disagree (1)” to “strongly agree (5).”

A meticulous in-person survey was conducted among employees in the hospitality sector of Northern Vietnam, a region notable for hosting three of the eight UNESCO World Heritage sites [17]. Following the data collection, a detailed review ensured the accuracy of the gathered data. The dataset was subjected to rigorous analysis utilizing SPSS version 25 and SmartPLS 3.2.9, ensuring that the AVE, CR, and CA adhered to recommended thresholds. The AVE analysis confirmed that a substantial part of the variance in the constructs is explained by their respective latent variables, not by measurement error, thereby establishing convergent validity. Multicollinearity was examined using the VIF, which verified that the constructs were distinct and not excessively intercorrelated. Additionally, discriminant validity was examined through the Fornell-Larcker criterion and the Heterotrait-Monotrait (HTMT) ratio technique. The study culminated in the creation of a PLS-SEM model that was used to test the hypotheses and clarify the correlations among the constructs involved in the research.

## Limitations

Our study examining PSS and JP within the Vietnamese hospitality industry has several limitations. First, the cultural nuances of Vietnam, with their emphasis on hierarchical respect and collective well-being, may uniquely influence employees’ perceptions of supervisory support, potentially differing from other cultural or industry contexts where individualism and self-direction are more prevalent. Additionally, the rapidly growing economic context but relatively low wage structure in Vietnam’s hospitality industry may bias perceptions of job satisfaction and performance relative to industries in more developed economies or industries such as dairy industry, canning industry, and bread, cake, and pastry idustry. These context-specific factors may limit the generalizability of our findings to other contexts or industries without further contextual adjustment or comparative research.

## Ethics Statement

Participants were fully informed about the research aims and scope before participating, and their informed agreement was obtained. They remain anonymous, with no possibility of being contacted. Approval from the Institutional Review Board (IRB) was not required for this research. Participation was entirely voluntary, and the survey's cover letter guaranteed participant confidentiality, stating that participation implied consent. The study excluded juveniles and adhered to the principles of the Helsinki Declaration. To ensure security, all data collected is anonymized, personally identifiable information is removed, and a unique code replaces any identifying information, maintaining the privacy and security of all participant adhered to ethical research standards.

## Credit Author Statement

**Bao Han Le**: Conceptualization, Supervision, Methodology, Software, Data curation, Investigation, review & editing; **Cong Hiep Duong**: Conceptualization, Methodology, Software, Validation, Formal analysis, Visualization; **Gam Thi Nguyen**: Conceptualization, Methodology, Software, Data curation, Investigation, Original draft preparation, Writing.

## Data Availability

Mendeley DataData_PSS_Job performance (Original data)
